# Gallstone ileus: A rare cause of small intestine obstruction

**DOI:** 10.1002/ccr3.4924

**Published:** 2021-11-06

**Authors:** Francesk Mulita, Levan Tchabashvili, Dimitrios Bousis, Dimitrios Kehagias, Charalampos Kaplanis, Elias Liolis, Ioannis Perdikaris, Fotios Iliopoulos, Georgios‐Ioannis Verras, Konstantinos Bouchagier

**Affiliations:** ^1^ Department of General Surgery General University Hospital of Patras Patras Greece; ^2^ Department of Internal Medicine General University Hospital of Patras Patras Greece

**Keywords:** cholelithiasis, gallstone ileus, small bowel obstruction

## Abstract

Gallstone ileus is a very rare cause of mechanical bowel obstruction with often‐delayed presentation and nonspecific symptoms. Aerobilia is found in approximately 50% of patients with gallstone ileus.

## Graphical abstract

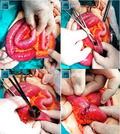

## 1 CASE DESCRIPTION

**FIGURE 1:**
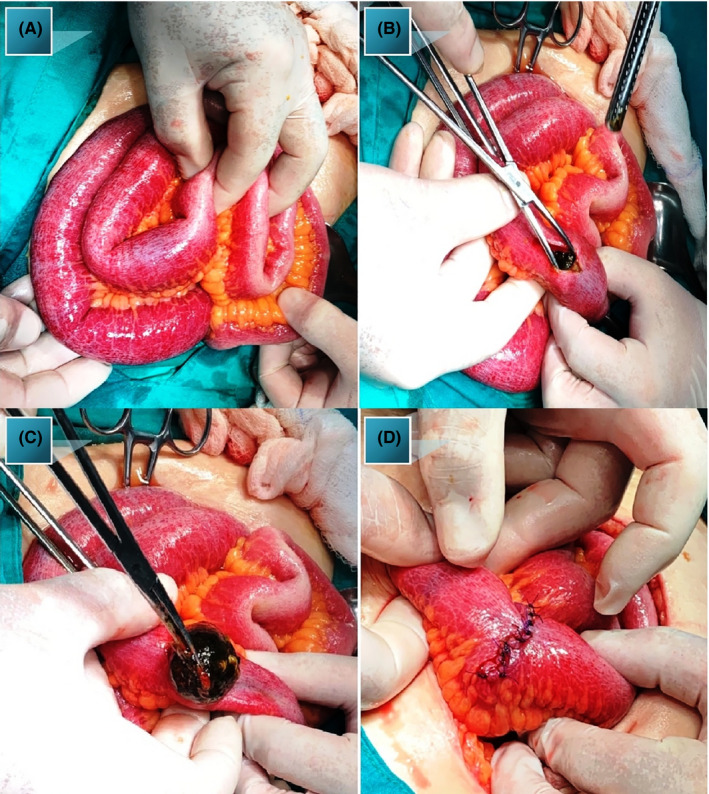
(A) Impacted gallstone. (B, C) Small bowel enterotomy for removal of the impacted gallstone. (D) The enterotomy was closed transversely

A 77‐year‐old woman was admitted to our emergency department (ED) complaining of abdominal distension, vomiting, and nausea. During physical examination, normal bowel sounds were auscultated, and there were no signs of abdominal flatulence or tenderness. Her routine blood tests were normal. However, her C reactive protein was elevated. Abdominal x‐rays revealed dilated small bowel as well as air‐fluid levels. A computed tomography (CT) was performed and showed aerobilila and a large 5.1 cm gallstone lodged in the small intestine. The patient was resuscitated with intravenous fluids and underwent emergency surgery. Intraoperative findings noted small bowel obstruction with the transition point at 70 cm from the ileocaecal valve caused by a large gallstone obstructing the lumen (Figure 1A). A longitudinal 3 cm enterotomy was made proximal to the distal gallstone (Figure 1B). The stone was removed, and the enterotomy was closed transversely (Figure 1C, D).

Gallstone ileus develops in less than 0.5% of patients with cholelithiasis and accounts for less than 5% of nonstrangulating mechanical small bowel obstructions. Patients have nonspecific symptoms and the diagnosis is often delayed since symptoms may be intermittent and investigations may fail to identify the cause of the obstruction. The majority of reported cases of obstruction demonstrate a gallstone larger than 20 mm in diameter.^1^ If a clinician has a clinical suspicion of gallstone ileus but the patient has negative radiograph findings, a computed tomography (CT) scan should be performed. Aerobilia is found in approximately 50% of patients.^2^

## CONFLICTS OF INTEREST

There are no conflicts of interest to declare.

## AUTHOR CONTRIBUTIONS

FM, DK, LT, G‐IV, IP, FI, CK, and EL contributed to the clinical data collection and prepared the case report. FM, LT, and EL contributed to the design of the case report presentation and performed the final revision of the manuscript.

## CONSENT

A written informed consent was obtained from the patient for publication of this case report.

## Data Availability

Data available on request from the authors.
